# New Graduate Nurses' Experiences and Challenges during a One-Year Clinical Rotation Programme in the Volta Region of Ghana: Implications for Nursing Management and Nursing Workforce Retention

**DOI:** 10.1155/2022/5023419

**Published:** 2022-12-20

**Authors:** Peter Adatara, George Sedinam Boni

**Affiliations:** ^1^Department of Nursing, University of Health and Allied Sciences, PMB, 31 Ho, Ghana; ^2^Accident and Emergency Department, Ho Teaching Hospital, Ho, Ghana

## Abstract

**Background:**

To help improve the standards and quality of nursing practice, newly graduated nurses and midwives are required after passing the Nursing and Midwifery Council's Licensing Examination to undertake a one-year mandatory clinical rotation at health facilities before registration. However, there is a lack of scientific studies to explore new graduate nurses' experiences and challenges during the one-year mandatory clinical rotation programme.

**Aim:**

This study is aimed at exploring newly qualified nurses' experiences and challenges during the one-year mandatory clinical rotation in health facilities in the Volta Region of Ghana.

**Methods:**

This qualitative exploratory study used a combination of focus group discussions (FGDs) and individual interviews. Thirty (30) newly qualified nurses were selected from two secondary and one tertiary health facilities, where newly qualified nurses are posted for a clinical rotation programmes to participate in the study. A total of two focus group discussions (FGDs) were conducted, comprising five (5) members in each group. Moreover, twenty (20) newly qualified nurses were interviewed individually, and their data were added to what was obtained from the focus group discussions. This study adopted the thematic analysis approach to analyze the data.

**Results:**

The results of this study showed that newly qualified nurses experienced the following during the one-year clinical rotation programme: acquisition of more knowledge and competencies to assume professional nursing roles; lack of knowledge of the objectives of the clinical rotation programme by hospital staff; experience of frustration during the clinical rotation programme; inadequate supervision and support; accommodation and financial challenges.

**Conclusion:**

The study revealed important challenges experienced by new graduate nurses during the clinical rotation programme, including hospital staff's lack of knowledge regarding the objectives of the rotation programme, inadequate supervision and support, and accommodation and financial challenges. There is a need for the Nursing and Midwifery Council to develop guidelines for the one-year mandatory clinical rotation for newly graduated nurses and midwives to make the programme more effective.

## 1. Background

Research showed that mandatory clinical rotation programmes have been introduced and implemented by many countries in the world for newly qualified nurses and midwives who had completed the prescribed academic and professional studies as a way of preparing them to assume professional roles [1, 2]. Empirical evidences revealed that newly graduated nurses and midwives, transitioning from universities and other training institutions in developed and developing countries to practice in healthcare settings, remain challenged, stress, and emotionally exhausted [3, 4] as they strive to deliver safe nursing care amidst heavy workloads and increased accountability and responsibility for their patient care [5]. Due to the challenges of newly qualified nurses transitioning from university to practice, mandatory clinical rotation programmes are transitional programmes and interventions organized by professional Nursing and Midwifery Councils in many countries to address negative experiences that have been found to result in feelings of heightened work stress for up to one year after graduation, with contributory factors including poor work environments, poor clinical supervisors, and poor nurse-doctor relation [6].

Clinical rotation is a transition from being a student nurse to becoming a full-fledged nurse within the first year of professional nursing practice [7]. An effective clinical rotation programme allows newly trained nurses to familiarise themselves with the working environment and organizational culture and thus promote the development of clinical proficiency, support professional development, and improve new graduate nurse retention [8]. Studies indicate that clinical rotation programmes enable newly qualified nurses and midwives to apply the theory of nursing, facilitating the integration of theoretical knowledge and practical skills in the clinical setting, which becomes the art and science of nursing [9, 10]. A study from Australia indicates that all new graduate nurses are required to undertake a 12-month transitional support programme to enable them to acquire the knowledge, attitudes, and behaviours to perform effectively and adjust to their work surroundings [6]. Similarly, in South Africa, impirical evidence shows that the South African Nursing Council (SANC) in 2004 introduced a one-year mandatory clinical rotation programme for all newly qualified nurses after graduation, and before the graduate is registered as a professional nurse with the South African Nursing Council [11]. According to SANC, the main objective for the introduction of community service for health professionals was to assist nurses and other health professionals to develop further practical skills, knowledge, critical thinking, and professional behaviour during the period of compulsory community service [12, 13].

Despite the importance of the one-year mandatory clinical rotation programme for newly qualified nurses and midwives, previous studies suggested that these programmes could only be effective in achieving their objectives if there were preceptors or clinical supervisors in the clinical setting to supervise and guide the newly qualified nurses in the transition process of becoming fully-fledged professional nurses and midwives [14, 15]. Studies in other countries indicate that newly qualified nurses and midwives experience a myriad of challenges, such as a lack of preceptors and mentors to supervise newly qualified nurses, which underscores the purpose of clinical rotation programmes [6, 16] . In addition, clinical rotation experiences often do not match the values and ideals initially perceived before the start of clinical rotation for newly qualified nurses and midwives [17]. Studies have indicated that falling standard nursing care could be addressed if professional regulatory bodies such as the Nursing and Midwifery Councils could implement effective mandatory clinical rotation programmes for newly qualified nurses and midwives to prepare them for the field of work [1].

Similarly, in Ghana, all nurses and midwives who graduated from universities and nursing training colleges and passed the Nursing and Midwifery Council's Licensing Examinations are required by the policy of the Ministry of Health to undertake the one-year mandatory clinical rotation programme before they could be employed. However, since the introduction of the one-year mandatory clinical rotation for more than a decade, it has been observed that there is no scientific study on the experiences and challenges of newly qualified nurses and midwives during the one-year mandatory clinical rotation to ascertain whether the programme has achieved its desired objectives in the country. Understanding the experiences and challenges of newly qualified nurses and midwives regarding the clinical rotation programme is critical in achieving the goals and objectives of the programme. The findings of the study would also inform stakeholders, such as the Nursing and Midwifery Council (NMC) of Ghana, the Ghana Health Service, and various teaching hospitals, on the measures to put in place to address the challenges that newly qualified nurses and midwives experience during the period. This study is aimed at exploring newly qualified nurses' experiences and challenges during a one-year clinical rotation programme in the Volta Region of Ghana.

## 2. Methods

### 2.1. Study Design and Setting

This study employed a qualitative exploratory approach to explore newly qualified nurses' experiences during a one-year mandatory clinical rotation at various health facilities in the Volta Region of Ghana. The qualitative exploratory design was chosen because it allowed the researcher to gain a better understanding of newly qualified nurses' experiences and challenges during the clinical rotation nurses programme [18]. It allowed researchers to provide rich descriptions of participant experiences during the one-year clinical rotation that are not readily accessible through more quantitative means [19].

The study was carried out at two secondary and one tertiary health facilities in the Volta Region of Ghana, where newly qualified nurses and midwives were posted to undertake a one-year mandatory clinical rotation programme. These health facilities were purposefully selected for the study because of the fact that these health facilities are referral health facilities in the Volta Region with qualified health staff with the necessary medical equipment suitable for the training of newly qualified nurses and midwives. These facilities were also chosen for the study because of the availability of newly qualified nurses for semistructured and focused group interviews.

### 2.2. Study Participants and Sample Size

The participants for this study included thirty (30) newly qualified nurses and midwives who had completed the one-year mandatory clinical rotation and were selected from five health facilities in the Ho Municipality in the Volta Region of Ghana. The inclusion criteria were newly qualified nurses and midwives who had a bachelor's degree in nursing or midwifery, and who had completed the clinical rotation as required by the Nursing and Midwifery Council of Ghana. Newly qualified nurses and midwives whose qualifications were diploma certificates were excluded from this study. Moreover, nurses and midwives who did not complete the one-year rotation programme were excluded.

### 2.3. Data Collection Process

This qualitative exploratory study adopted a combination of focus group discussion and individual interviews to explore newly qualified nurses' experiences and challenges during the one-year clinical rotation programme. Thus, data were collected through semistructured individual interviews and focus group discussions (FGDs). Semistructured interviews were conducted to collect data from twenty (20) newly qualified nurses and midwives who completed the one-year mandatory clinical rotation programme selected from two secondary and one tertiary health facilities in the Volta Region of Ghana. The interviews were all conducted in English by the first author (P. A), who has a PhD with many years of qualitative research experience. All participants were asked the same questions during the individual semistructured interviews. Each interview lasted between 45 and 60 minutes.

A total of two (2) FGDs were conducted, with each FGD comprising five (5) participants. Each group was homogeneous, comprising newly qualified nurses who had completed their one-year mandatory clinical rotation programme. The FGDs were framed by semistructured questions to gain an informed understanding of the participants' experiences regarding the clinical rotation programme for newly qualified nurses. The first author conducted all individual interviews and the FGDs. The FGDs were conducted after working hours when participants were not on duty. The interviews were audio-recorded. Each FGD lasted between 45 and 60 minutes. The researcher engaged with participants by posing questions in a neutral manner, listening attentively to participants' responses, and asking follow-up questions using probes based on their responses. The questions that were asked with probes were asked as follows:

- What were your experiences during the one-year clinical rotation at the health facility?
- What support did you receive from the health facility during the one-year clinical rotation
- Did the health facility sufficiently prepare or orient you for the clinical rotation programme?
- What challenges did you encounter during your clinical rotation?
- How did you overcome (if any) the challenges you encountered during your clinical rotation?
- Did you have a mentor in the wards?
- How was your relationship with your mentor?
- How was your relationship with the staff members of the health facility?
- What did you gain as a professional from the clinical rotation programme?
- Do you think the clinical rotation programme year is of any value to newly qualified nurses?
- What suggestions would you make to help improve the clinical rotation programme for newly qualified nurses in Ghana?

Probes were used when there was a need to clarify the information given by participants on their experiences. Interviews were conducted in the English language. All discussions were recorded using a digital voice recorder after which they were transcribed accordingly. Daily interviews were shared with other authors to review and provide feedback on the process. This interactive approach strengthened the data elicitation process. Interviews continued until data saturation was achieved. We also wrote comprehensive field notes daily. These field notes were incorporated into the data set. In all, 30 people were interviewed.

### 2.4. Data Analysis

Data were analyzed using inductive reflexive thematic analysis approach. The inductive reflexive thematic approach involves allowing the data to determine the themes of the study [20]. The inductive reflexive thematic analysis involves a reflexive, recursive engagement with the dataset, to produce a robust analysis [20]. The reflexive thematic analysis approach requires that the researcher attempts to make sense of the situation without imposing preexisting expectations on the phenomenon or setting under study. It involves reading through textual data and identifying and coding emergent themes within the data [20]. Six-step model proposed by Braun and Clarke [21] was used. After developing initial codes, themes were generated, reviewed, and defined. Data collection and analysis were done concurrently which allowed probing in subsequent interviews. The data were cleaned to get rid of all identifiable data. The first step was reading the transcripts to become familiar with the data. After that, the transcripts were read severally to have a deep understanding of participants' perspectives. They were then reviewed independently by three researchers with rich experience in qualitative research for accuracy and objectivity. Discussions among the researchers on the themes generated and ensured that the data were free of personal biases. The next step was that the data were coded using the NVivo version 12 software, and initial codes were generated from the coded data. The coding was done according to the themes of the research questions of this study. After generating many codes, the researchers searched for themes relevant to the research questions. Codes were then put together into themes. Initially, we identified eight themes. However, after discussions among the researchers, the two themes were later merged, resulting in five main themes. Thick verbatim quotations were also used to back up the findings of the study. The researchers met severally to analyze the emerging themes to ensure that it refected the true meanings of participants' perspectives.

### 2.5. Trustworthiness of the Study

Trustworthiness of this study was ensured by using the same interview guide to interview all participants in the study. The interviews were all conducted by the lead author, who has a PhD with several years of experience in qualitative research studies. Some of the participants were contacted later during data transcription and analysis for clarification and member checking. An audit trail was used to determine whether the conclusions, interpretations, and recommendations could be traced to the source of information/data. Field notes which were taken during data collection were used as back-ups during data transcription to verify participants' responses. To further enhance credibility of the findings, the researcher audiotaped all the interviews which were transcribed verbatim. The researcher was also reflexive during the data collection and data analysis process so that he did not influence the outcome of the research. Concurrent data collection and transcription ensured that emerging themes and subthemes were probed in subsequent interviews.

### 2.6. Ethics Approval and Consent to Participate

Ethical clearance for the study was obtained from the Ethical Review Committee of the University of Health and Allied Sciences (UHAS-REC A.2 [18] 18-19). Moreover, institutional approval from the hospital was sought before the start of data collection. Participants' information leaflets, which contained all information regarding the study before the participants gave consent, were explained to all participants. Participants were assured that they could withdraw from the study any time and that such a decision will not be used to victimise them when they seek healthcare or anything. After explaining the purpose of the study, participants were given informed consent forms which were duly filled in and signed by them. The semistructured interviews were conducted using their identity numbers instead of their names to ensure the confidentiality of participants.

## 3. Results

### 3.1. Social Demographic Characteristics of Participants

A total of thirty (30) newly qualified nurses and midwives participated in the study. The ages of the participants ranged from 24 to 29 years. The participants included seventeen (17) nurses and thirteen (13) midwives. Fourteen (14) of them were males while sixteen (16) of them were females. All participants had 12 months mandatory clinical rotation experience.

### 3.2. Themes Generated from the Data Analysis

Five (5) main themes concerning the experiences and challenges of rotation nurses and midwives were generated from the data analysis:

- Acquisition of knowledge and competencies during clinical rotation programme
- A lack of knowledge of the objectives of the clinical rotation programme by hospital staff
- Experience of frustration during the clinical rotation programme
- Inadequate supervision and support
- Accommodation and financial challenges

### 3.3. Theme 1: Acquisition of Knowledge and Competencies during the Clinical Rotation

One of the main themes that emerged from the data analysis was the fact that the participants acquired new skills during the clinical rotation that sought to prepare them to assume professional roles. Participants indicated that although the one-year mandatory clinical rotation programme was tedious and frustrating, it prepared them for professional roles in nursing and midwifery. The following are illustrative quotes from the respondents to support their claim:

“Generally, my clinical rotation at the said facility has been awesome in that I got full exposure to various disease conditions and their management. I was given the privilege to have hands-on experience in most of the nursing procedures, however, it was very difficult for me during the start of the internship programme”. (PA 01)

“The one-year mandatory clinical rotation programme is really helpful. It has prepared me to assume and perform the role of a professional nurse when I am employed”. (PA 18).

“I have learned a lot from this one-year clinical rotation programme. The things I did not know and the nursing procedures I could not perform when I was a student for four years at the university, I can now do them successfully”. (PA 13).

Participants indicated that apart from learning new things from the clinical rotation programme, it has also developed their confidence in performing of nursing procedures. The following are illustrative quotes from the respondents to support their claim:

“Now, I have confidence in myself when I am carrying out a procedure, unlike when I was a student. I see myself as a qualified nurse rather than someone who has just finished training because of the confidence I have developed in the ward during my clinical rotation”. (PA 15).

“The rotation gave a lot of confidence and exposure to almost every department a nurse could work in, and I think this has fully prepared me to fit into any ward when posted for practice”. (PA 20).

Participants also described the clinical rotation programme as an opportunity to polish and utilize the nursing care plan very well. They indicated that when they were students, they used the nursing care plan only for examination purposes but rarely used it well anytime they were on clinical practice placement. The following are illustrative quotes from the respondents to support their claim:

“One important thing I must mention is that I can now understand and utilize the nursing care plan very well when nursing patients and clients. I had thought that using the nursing care plan was difficult because we hardly used it well when we were students. However, now I enjoy using it”. (PA 19).

“One area I learnt much during this clinical rotation programme is the nursing care plan. I used not to like it when I was a student nurse. However, now I have used it not for just examination purposes but in real work”. (PA 20).

Moreover, some levels of leadership and managerial skills were acquired during the clinical rotation programme. Participants indicated that in some of the units they worked in the hospital, they had the opportunity to draw the duty roster and plan for the unit. These leadership and managerial skills they acquired from the units will help them perform in any nursing management position, as illustrated by these comments:

“One thing I also experienced during the clinical rotation and I was so excited about was some form of leadership and managerial skills I learnt in the wards. In some of the wards where I worked, I had the opportunity to draw a duty roster under the instruction and supervision of the nurse manager. This made me happy because if one day I become a nurse manager I won't suffer to do that”. (PA15).

“I must also emphasize that the clinical rotation programme has not only taught me clinical procedures and experience but also managerial and leadership skills. I was asked by one of the nurse managers to plan and make a requisition for the ward. I was able to do it under her supervision”. (PA09).

### 3.4. Theme 2: The Hospital Staff Did Not Know the Objectives of the Clinical Rotation Programme

Participants indicated during the interviews that some of the nursing staff in the wards did not clearly understand the objectives of the one-year mandatory clinical rotation programme for newly qualified registered nurses and midwives, as captured in the following quotes:

“As for me, it appears the ward nurses do not clearly understand what is expected of us as rotation nurses. Therefore, they just look like people who do not understand why we are there in the wards”. (PA10).

“I was surprised when some of the nurses in the wards asked me why I was in the ward when I am not a qualified nurse yet”. (PA01).

Participants reported that nursing staff in the wards did not clearly understand the objectives and roles of the clinical rotation programme because, according to them, the nursing professional regulatory body, the Nursing and Midwifery Council of Ghana, did not state any clear objectives to be achieved within the clinical rotation programme.

“I think the nurses do not understand the objectives and roles of the clinical rotation programme because the Nursing and Midwifery Council of Ghana did not give us any learning objectives for the clinical programme”. (PA09).

“As for me, I do not blame the nurses in the wards for not understanding what we do in the hospital. I blame the Nursing and Midwifery Council of Ghana because they did not even give us any letter containing what is expected of us as rotation nurses”. (PA02).

Some of the participants indicated that although the Nursing and Midwifery Council of Ghana regulates the training and practice of nursing and midwifery, it was rather the Ghana National Services Secretariat that does not understand nurses' work before posting them to various facilities. These were some of their comments:

“What the Nursing and Midwifery Council of Ghana does is not right. The Council allows the personnel of the Ghana National Services Secretariat, who are not nurses and do not understand the objectives and work of the clinical rotation programme, to post us without any rotation programme objectives”. (PA05).

“What is confusing to us and the nurses in the ward is the fact that the rotation nurses were initially posted by the Nursing and Midwifery Council of Ghana to undergo the one-year mandatory clinical rotation, but we were posted by the Ghana National Services Secretariat, which created the confusion”. (PA08)

Most participants reported that because nurses do not clearly understand the objectives of the clinical rotation programme, they do not exactly know what nursing procedures they should be allowed to perform. Therefore, in some of the units, the nurses decided to allow them to perform the duties of a qualified nurse, while in some units, they were asking them to work like students.

“I must say that because the nurses do not know the objectives of the clinical rotation programme, in some of the wards, the nurses allowed us to work like qualified nurses while in some of the units, we were only allowed to work like students”. (PA15).

“Sometimes, I was not permitted to develop my sense of initiative by some staff nurses: I was told many times that the classroom knowledge is different from what is being done here. Don't come and show us book-long things”. (PA11).

### 3.5. Theme 3: Newly Qualified Nurses Experienced Frustration during the Clinical Rotation Programme

The participants described the one-year mandatory clinical rotation programme as frustrating. They indicated that it was much easier and less stressful anytime they were put into clinical practice compared to the clinical rotation, where they worked almost every day.

“It is more difficult than when we were students. We now have to work almost every day of the week. We were only allowed to take one day off a week. It is really difficult”. (PA 14).

“I must admit that when I was a student, I never thought working in the wards could be difficult this way. It has been difficult for me since I started working as a rotation nurse”. (PA12).

Participants believed that the clinical rotation was uninteresting because it was unstructured. They reported that they would not have complained about the nature of nursing practice if they had a well-structured rotation programme and support from staff.

“It is rather unfortunately that we are complaining about the nature of work. The clinical rotation programme is not well structured for us to know that this is how we are going to start, and this is where we will end. However, there is nothing like that here”. (PA 19).

“We are just there, confused and tired. There is no structure in place. The ward nurses are now the ones telling us what to do daily, making work difficult for us because they can ask us to do anything, whether it is related to nursing or not. We will be forced to do it”. (PA16).

Participants reported that other things that made them frustrated with the clinical rotation were that some of the staff nurses in some health facilities did not respect them and did not value their contribution to the wards towards the care of patients. They felt that they were not needed in the wards.

“I get frustrated almost every day I go to work. It appears the nurses do not need us around in the wards because they do not respect us, and they never accept suggestions from us. Therefore, we are just sitting there like we are still students”. (PA 01).

“My dignity as a human was not respected by some of the nursing staff as they shouted at me, insulted me just because I am a rotation nurse”. (PA 10).

Participants indicated that maltreatment and disrespect were common among the nurses who were diploma certificate holders. This category of nurses in the wards felt that although the rotation nurses were first-degree holders, they did not know the clinical work. Participants believed that the nurses' statements that they did not know the clinical work resulted from envy and not facts.

“I must say that we were mostly abused and insulted by the nurses in the wards who are diploma holders and not bachelor's degree holders. They were the ones who kept on saying that we are degree nurses, and yet we did not know anything. We know they are just saying it out of envy, not because we do not know the job”. (PA 03).

“We are frustrated and insulted every day in the wards for no fault of ours by the nurses who do not have a bachelor's degree. They will always look for every single opportunity to insult and embarrass us every day”. (PA 06).

Participants reported that due to the insults and embarrassments from the ward nurses, they were always working under intense pressure, being cautious not to make mistakes that could be used to them by the staff nurses.

“It is stressful and frustrating as a degree rotation nurse. We are always trying to work hard and be cautious not to make mistakes that could be used against us. Even with that effort, they still insult us. (PA 13).

“We are careful about whatever we do in the wards as rotation nurses because of the constant criticism and embarrassment from the staff nurses”. (PA 17).

“We cannot even take our own initiative in the ward because if you try it and you do not get it right, they will use it to you and insult you”. (PA 20).

Although the participants described some of the nurses as being disrespectful, they indicated that some of the nurses in the wards were very helpful and were always there to talk to them and help them with whatever they did in the wards.

“I must say that not all the nurses in the wards were bad toward us. Some of them were nice and supportive during the clinical rotation programme and need to be commended”. (PA 18).

“Honestly, there were some of the units I worked during rotation that I enjoyed so much. The nursing staff were caring, helpful, and always ready to assist me in any procedure that I wanted to carry out that I was not too comfortable with. It was really amazing working in those units”. (PA 19).

### 3.6. Theme 4: Inadequate Supervision and Support

Supervision and mentorship are important in successfully completing the one-year mandatory clinical rotation programme. Participants reported that they experienced inadequate supervision and mentorship during the clinical rotation programme.

“Supervision was inadequate. I was left on my own to do whatever I wanted. In some of the wards, I was only running a shift with staff nurses who completed their training two years ago and were not experienced themselves”. (PA02).

“Very few of the staff in the hospital were supportive while the majority perceived me as a threat and did not want to teach me anything. Preceptors were not available for supervising the learning activity in the wards”. (PA15).

The participants reported that anytime they encountered problems in the ward, there was no one to talk to for assistance.

“It was very bad for me because no one was assigned to me as my preceptor or supervisor in the various wards where I did my clinical rotation. Therefore, anytime I had a problem and wanted assistance, there was nobody to guide me. (PA19).

“I felt nobody wanted to supervise me in the wards because there were instances I asked for help from the ward staff but there was nobody to listen to me. I think this was happening to me because there was no person assigned to me as a supervisor”. (PA05).

Participants indicated that they were told that the nurse managers (ward-in-charges) were their supervisors and preceptors, but they were too busy and could not help them.

“The Director told us of Nursing of the hospital that ward-incharges were preceptors and supervisors in the wards, but the nurse managers were too busy all the time and could not supervise us”. (PA08).

“As a matter of fact, the nurse managers were too busy at all times. There were instances during my rotation, I needed guidance from the nurse manager who was supposed to be a supervisor, but he/she was attending a meeting outside the ward”. (PA20).

### 3.7. Theme 6: Accommodation and Financial Challenges

Rotation nurses reported accommodation and financial challenges, which created problems for them during the clinical rotation programme. They indicated that many of them faced accommodation and financial challenges because they were not provided accommodation for the clinical rotation programme.

“One of the major challenges we encountered when starting the clinical rotation programme was accommodation. We were not provided accommodation nor given enough time to prepare to start the programme”. (PA14).

“I had accommodation issues when I started the clinical rotation programme. I did not have a room when I reported to work, so I was putting up with a friend for some time before I could get an apartment on my own”. (PA20).

Participants indicated that apart from the accommodation problem, they also encountered financial challenges, which affected them psychologically.

“I must say that when we started the clinical rotation, we were never paid the National Service Allowances paid by the National Service Secretariat for almost four months, and yet we were going to work. And some of us, our parents were not earning income to support us. It really caused so many psychological stresses at the time”. (PA17).

“As for me, I almost stopped the clinical rotation programme because at a point I did not have any money with me, and the National Service Secretariat was not paying us our allowances for three months”. (PA09).

The accommodation and financial challenges made participants engaged in other part-time jobs, such as teaching in senior high schools around the town to make life easy. Other rotation nurses were engaged in selling “provisions” to earn income for their upkeep during the clinical rotation.

“My main challenge was the delay in paying our allowances by the National Service Secretariat, so I had to resort to some part-time teaching to be able to survive while waiting the allowance”. (PA10).

“I had to engage in assisting a woman in selling her provisions in her shop to survive. There was nothing I could do to earn a living. All these things affected me psychologically, and my concentration on the programme was very low”. (PA13).

## 4. Discussion

This study, which was the first of its kind in Ghana, was conducted to explore newly qualified nurses' experiences and challenges during the one-year mandatory Nursing and Midwifery Council clinical rotation before employment in healthcare facilities in Ghana. The results of this study showed that newly qualified nurses gained more knowledge and clinical competencies within the one-year mandatory clinical rotation programme, which has prepared them to assume professional nursing roles in healthcare facilities. The finding confirms a previous study which indicated that newly graduated nurses benefited from the clinical programme because it made them professionally matured and assisted in boosting the readiness of new graduate nurses' transition to become skilled professional nurses [22]. It also serves as an opportunity for nurses to learn to apply the theory of nursing, facilitating the integration of theoretical knowledge and practical skills in the clinical setting, which becomes the art and science of nursing [23]. It was reported that an effective clinical rotation programme for newly qualified nurses allows beneficiaries of the programme to familiarise themselves with the working and organizational culture of the health facilities and ease them through the transition process of becoming qualified practitioners [3]. The above findings imply that there is a need for a properly organized clinical rotation programme for newly qualified nurses to enhance their skills and competencies in the field of work.

Moreover, the results of this study showed that some of the supervisors and nursing staff of the health facilities where newly qualified nurses undertook the one-year mandatory clinical rotation did not clearly know or understand the objectives of programme. This made it difficult for the nursing staff and supervisors in the hospital to provide the necessary guidance and assistance to the newly qualified nurses on a clinical rotation to achieve the objectives of the programme. Our finding is supported by previous studies, which indicated that the expectations of newly qualified nurses on clinical rotation were not met as nursing staff in some health facilities where newly qualified nurses are posted for the clinical rotation programmes were sometimes not aware of the learning objectives of the rotation programme [24, 25]. Moreover, one of the major findings of this study showed that newly qualified nurses experienced frustration during the rotation programme. The lack of knowledge of nursing staff and supervisors regarding the learning objectives for the clinical rotation programme and the unstructured nature of the programme by the Nursing and Midwifery Council is a major hindrance to the success of the programme. This practice has often left the beneficiaries of the programme frustrated and stranded in health facilities where newly qualified nurses are posted to the programme. This clearly undermines the purpose of the one-year clinical rotation programme [3]. These findings call for a well-structured clinical rotation programme with learning objectives to achieve the objectives of the clinical rotation programme for newly qualified nurses.

Additionally, it was reported by newly qualified nurses on the one-year mandatory clinical rotation that they experienced inadequate supervision and support during the clinical rotation programme. It was revealed that the lack of proper and effective supervision and support for newly qualified nurses resulted in inadequate staff, which created a heavy workload on the staff in the hospital. This finding is supported by a previous study which reported that newly qualified nurses on mandatory clinical rotation programmes lacked clinical tutorial support and guidance from experienced staff in the wards due to a shortage of personnel which limited the opportunities for proper teaching and guidance in the health facilities [26]. Similarly, a study conducted to explore the experiences of newly qualified nurses on clinical internships indicated a shortage of staff affected the newly qualified nurses on clinical rotation from acquiring the requisite competencies during the clinical rotation programme to assuming professional nursing roles when employed [6]. The finding implies that there is a need for health facilities where newly qualified nurses are posted for clinical rotation to put in place measures such as a duty roster which allows experienced nurses to run shifts with newly qualified nurses to provide them with the needed supervision, mentorship, and guidance to acquire the requisite skills to assume professional roles.

Accommodation and financial challenges were reported as some of the significant challenges experienced by newly qualified during the one-year mandatory clinical rotation. It came to light that newly qualified nurses on clinical rotation rented their own accommodation for the one-year mandatory clinical rotation programme. This created problems for most of them as they were not paid allowances regularly. It was further revealed that some of them had to borrow money from friends to pay for accommodation, feeding, transportation, and utility bills. Similarly, a study carried out in South Africa reported that newly qualified nurses on clinical rotation delayed in accepting a posting because of accommodation and financial challenges [24]. Generally, accommodation challenges and delays in the payment of allowances to newly qualified nurses have been major challenges among newly qualified healthcare staff that need to be looked at to ensure the welfare of newly qualified health staff on the clinical rotation programmes. There is a need for the Ghana Health Service and the health facilities to arrange for hostel accommodation and advance small loans to these nurses to enable them to undergo the clinical rotation smoothly.

### 4.1. Limitations of the Study

This study was conducted among newly qualified nurses with one-year clinical rotation experience from only one region out of the sixteen regions in Ghana. The views of the newly qualified nurses and midwives in other regions of Ghana were not explored. This limited the ability to generalize the findings of our study. Another limitation of this study was that the population for our study was limited to the experiences of only newly qualified nurses who graduated from tertiary institutions in Ghana. We could not explore the experiences of newly qualified nurses who completed nontertiary institutions or such nursing training colleges to have a clearer and broader understanding of the experiences of newly qualified nurses regarding the one-year clinical rotation programme. In view of the above, we recommend further studies to be conducted to explore the experiences of both nurses and midwives who graduated from universities and training colleges to have a broader understanding of the issues surrounding the prelicensure rotation programme for nurses and midwives in Ghana. Moreover, the small sample size for this study, which was thirty (30) newly qualified nurses who undertook a one-year clinical rotation programme, could not be a representative of the experiences of more than a thousand newly qualified nurses who took part in the clinical rotation programme within the period in Ghana. However, this study provided an in-depth understanding of the experiences of newly qualified nurses regarding the one-year clinical rotation in Ghana.

## 5. Conclusion

As the first of its kind to be conducted in Ghana, the study highlighted some positive experiences of newly qualified nurses during the clinical rotation as they acquired more knowledge and competencies. It also came to light in this study that newly qualified nurses on clinical rotatation faced challenges such as lack of a clear understanding by nursing staff regarding the objectives of the clinical rotation programme for newly qualified nurses, inadequate supervision and support by staff, and accommodation and financial challenges. There is a need for a well-structured clinical rotation programme with well-defined roles for nurses and midwives on clinical rotation programme.

### 5.1. Implications for Nursing Practices, Management, and Policy

- The findings of this study indicate that for the one-year clinical rotation programme to achieve its objective of facilitating the acquisition of skills by newly qualified nurses and midwives to assume professional roles, there is a need for stakeholders such as the Nursing and Midwifery Council of Ghana, Ghana National Service Secretariat, and the Ghana Health Service to come out with a structure for the one-year mandatory clinical rotation programme with clear programme objectives and well-defined roles to enable hospital staff to supervise newly qualified nurses and midwives on rotation
- A comprehensive job description of rotation nurses and handover policy document could be developed and made accessible to newly qualified nurses on clinical rotation programme. The policy document should contain administrative procedures, health facility policies, and the routine/schedule of the ward they are assigned to. These would ease the frustration of newly qualified nurses on the clinical rotation programme
- Participants of this study have stated that one of their biggest challenges was inadequate support and mentorship. Purposefully, linking the newly qualified nurses on clinical rotation to experienced nurses or mentors may help them to feel less isolated and could provide them access to guidance when they need it. A trained preceptor or supervisor from each of the units in the health facility must be assigned to mentor newly qualified nurses and midwives on rotation
- Finally, it is recommended that the National Service Scheme always pay the monthly allowances of rotation nurses and midwives on time to enable them to cater for their basic needs such as food, clothing, and shelter

If these measures highlighted above are implemented, they will go a long way to improve the clinical rotation programme for newly qualified nurses and midwives in Ghana.

- It is recommended that further studies should be conducted to explore the experiences of both nurses and midwives who graduated from universities and training colleges to have a broader understanding of the issues surrounding the prelicensure rotation programme for nurses and midwives in Ghana. A survey study covers a large number of rotation nurses and midwives who undertook a one-year mandatory clinical rotation programme in health facilities from all regions of Ghana to gain a clear understanding of the challenges rotation nurses encounter in health facilities in Ghana

## Data Availability

Data is available upon request to the corresponding author.
